# Impacts of Informant Replacement in Industry‐Sponsored Alzheimer’s Disease Clinical Trials

**DOI:** 10.1002/alz.093015

**Published:** 2025-01-09

**Authors:** Mikaela K Nishida, Michelle M Nuño, Joshua D Grill, Daniel L Gillen

**Affiliations:** ^1^ University of California, Irvine, Irvine, CA USA; ^2^ University of Southern California, Los Angeles, CA USA; ^3^ Institute for Memory Impairments and Neurological Disorders, University of California, Irvine, Irvine, CA USA

## Abstract

**Background:**

In Alzheimer’s disease (AD) clinical trials, participants must enroll with a study partner informant who accompanies them to visits and completes validated instruments. Mid‐trial informant replacement (IR) has been found to impact academic trial results. We hypothesized that a similar impact would be observed in industry‐sponsored trials.

**Method:**

We conducted a retrospective analysis of two industry‐sponsored AD clinical trials testing semagacestat in mild‐to‐moderate AD. We assessed the relationship between IR and Alzheimer’s Disease Cooperative Study‐Activities of Daily Living (ADCS‐ADL) score between visits using generalized estimating equations (GEE). The outcomes of interest were mean change in ADCS‐ADL between successive visits and the mean absolute change in ADCS‐ADL between successive visits, which was used to assess variability. Both models were adjusted for a priori‐specified potential confounding variables including participant sex, age, informant type, trial, time, and previous ADCS‐ADL measurement. To analyze impacts on a primary outcome measure, we used an ANCOVA model to estimate the association between IR and 76‐week change from baseline in ADCS‐ADL, where we adjusted for participant sex, age, informant type, trial, and baseline ADCS‐ADL measurement. We conducted an F‐test to compare the variances of this change.

**Result:**

Among N = 2642 randomized participants, 72 (∼3%) of participants experienced IR at least once with a total of 81 occurrences. For visits standardized to be three months apart, we estimated that the difference in the mean between‐visit change in ADCS‐ADL was approximately ‐1.41 points (95% CI: ‐3.52, 0.72; P = 0.194) and the difference in the mean between‐visit absolute change was approximately 1.85 points (95% CI: 0.22, 3.49; P = 0.026), comparing participants who experienced IR to similar participants who had stable informants. We did not estimate a statistically significant difference in week‐76 change from baseline ADCS‐ADL (Est. = ‐0.98, 95% CI: ‐6.02, 4.06; P = 0.702) or a significant ratio of variances (Est. = 1.09, 95% CI: 0.65, 2.17; P = 0.700) comparing this measure for participants with IR to those with stable informants.

**Conclusion:**

IR is associated with increased variability in ADCS‐ADL measurements between successive visits and should be considered when planning AD clinical trials.

